# Combination therapy with antibody‑drug conjugate RC48 (disitamab vedotin) and zimberelimab (PD‑1 inhibitor) successfully controlled recurrent HER2‑positive breast cancer resistant to trastuzumab emtansine: A case report

**DOI:** 10.3892/ol.2023.13945

**Published:** 2023-07-05

**Authors:** Shanmin Fan, Lianxiang He, Die Sang

**Affiliations:** 1 Department of Medical Oncology, Beijing Chaoyang District Sanhuan Cancer Hospital, Beijing 100022, P.R. China; 2 Medical Affairs Department, Guangzhou Gloria Bioscience Co., Ltd., Beijing 100005, P.R. China

**Keywords:** antibody-drug conjugate, disitamab vedotin, programmed cell death protein-1, zimberelimab, HER2-positive breast cancer

## Abstract

Options for later-line therapy are limited for patients with human epidermal growth factor receptor 2 (HER2)-positive breast cancer who have exhibited resistance to several systemic treatments. Antibody drug conjugates (ADCs) and immune checkpoint inhibitors are novel approaches for HER2-positive breast cancer, but few reports have been published regarding the efficacy of their combinations, particularly in patients with prior ADC failure. The present report describes a case of recurrent metastatic HER2-positive breast cancer, which responded poorly to several perioperative systemic therapies, including chemotherapies, HER2-targeted antibodies, small molecule inhibitors and trastuzumab emtansine (an ADC), along with post-surgical radiotherapy. Following failure of front-line therapies for recurrent cancer located in the chest wall, combination treatment with another HER2-targeted ADC, disitamab vedotin (120 mg), and zimberelimab (240 mg), a fully humanized anti-programmed cell death protein-1 (PD-1) antibody, administered intravenously every 2 weeks, was initiated. The tumor lesions improved slightly after two cycles of treatment and shrunk markedly, and almost disappeared at the end of the sixth cycle of therapy. The patient is still in remission at present. The present findings suggest the potential efficacy of HER2-targeted ADCs combined with PD-1 inhibitors for patients with HER2-positive breast cancer, including those resistant to prior HER2-targeted ADCs.

## Introduction

Upregulation of human epidermal growth factor receptor 2 (HER2) is very common in breast cancer, which makes it an important target for systemic treatment (1). In the last decade, HER2-targeted antibody-drug conjugates (ADCs) have been emerging agents for HER2-positive breast cancer (2). Trastuzumab emtansine (T-DM1) has been approved by the U.S. Food and Drug Administration not only for metastatic or recurrent disease, but also as a postoperative or adjuvant therapy for early stages of the disease (2). Disitamab vedotin (RC48) is a novel HER2-targeted ADC, the cytotoxic payload of which is the microtubule inhibitor monomethyl auristatin E (3). RC48 has been granted marketing approval as later-line treatment for locally advanced or metastatic HER2-positive gastric cancer by The National Medical Products Administration (NMPA) of China. RC48 has also exhibited promising efficacy in a phase I study for both HER2-positive and HER2-low breast cancer (4,5). The objective response rates (ORRs) were 31.4% (22/70) and 39.6% (19/48) in the HER2-positive and HER2-low populations, respectively. The median progression-free survival (PFS) times were 5.8 and 5.7 months in the HER2-positive and HER2-low populations, respectively.

Immune checkpoint inhibitors (ICIs), such as programmed cell death protein-1 (PD-1)/PD-1 ligand (PD-L1) inhibitors, have changed the treatment landscapes of various malignant tumors, which constitute the present mainstay of systemic anti-cancer therapies along with cytotoxic agents and targeted agents (6). PD-1/PD-L1 inhibitors are widely recommended for a variety of solid tumors, and pembrolizumab, with/without chemotherapy, is recommended for HER2-negative or immune biomarker positive (for example, microsatellite instability-high and mismatch repair deficient) patients with breast cancer, according to current breast cancer guidelines (7). Zimberelimab (also referred to as GLS-010 and AB122), is a novel fully humanized anti-PD-1 monoclonal antibody, which has exhibited marked antitumor activities for several tumors in an early clinical trial (8). In a phase II study, zimberelimab monotherapy was revealed to be associated with an ORR of 90.6% in Chinese patients with relapsed or refractory classical Hodgkin's lymphoma (9) and was thus approved by the NMPA (10). In phase II studies of previously treated advanced cervical cancer, zimberelimab monotherapy was reported to be associated with a higher ORR compared with pembrolizumab monotherapy (26.83 vs. 14.6%; non-head-to-head) in a PD-L1-positive [combined positive score (CPS) ≥1] population (11,12).

To the best of our knowledge, it remains unclear whether re-introduction of another ADC targeting the same molecule, with the same type of cytotoxic payload, is effective for ADC-treated HER2-positive breast cancer. In addition, there are few reports addressing the efficacy and safety of ADC combined with ICIs in recurrent or metastatic HER2-positive breast cancer. The present report describes a 44-year-old female patient with recurrent metastatic PD-L1-negative, HER2-positive breast cancer, whose disease recurred in the chest wall during adjuvant treatment with T-DM1 (Kadcyla^®^) within 6 months. The patient then received combination treatment (inetetamab, pyrotinib and vinorelbine), followed by anlotinib (tyrosine kinase inhibitor) monotherapy. Due to poor tumor response, the patient was subsequently switched to another ADC, RC48 in combination with zimberelimab. The recurrent tumor lesions in the chest wall almost disappeared after six cycles of combination therapy and remain in remission at present.

## Case report

In early March 2021, a 44-year-old, premenopausal female patient was referred to Sanhuan Cancer Hospital (Beijing, China) due to a suspected breast malignant tumor [Breast Imaging Reporting and Data System (BI-RADS) ultrasound category 4], with initial symptoms of a mass in their left breast, dermal edema (peau d'orange) and enlarged left axillary lymph nodes. Later in March 2021, at the same hospital, magnetic resonance imaging (MRI) confirmed that the left breast malignant tumor (multiple nodules; maximum size, 4.9×1.9 cm; BI-RADS category 5) was metastatic to the left supraclavicular, axillary and internal mammary lymph nodes. Subsequently, the patient was pathologically diagnosed with grade III invasive breast cancer by needle biopsy using hematoxylin and eosin staining and a Ventana Benchmark XT system (Roche Tissue Diagnostics), according to the manufacturer's instructions (Fig. S1A and B) (13). Automated immunohistochemistry was also performed on 4-µm whole-tissue sections using a Ventana Benchmark XT system according to the manufacturer's instructions (antibodies are listed in Table SI). Histopathological examination was performed and images captured using a light microscope (Nikon ECLIPSE 80i; Nikon Corporation). The immunohistochemistry (IHC) scores were as follows [according to the American Society of Clinical Oncology guidelines (14,15)]: Estrogen receptor (ER)+, 40% (Fig. S2A and B); progesterone receptor (PR)- (Fig. S3A and B); HER2 3+ (Fig. S4A and B); P53+, 95%; Ki-67 proliferation marker, 80%; and epidermal growth factor receptor (EGFR) 2+. The tumor node metastasis (TNM) classification was cT4N3M0, stage IIIc (American Joint Committee on Cancer, 8th edition) (16).

**Figure 1.**
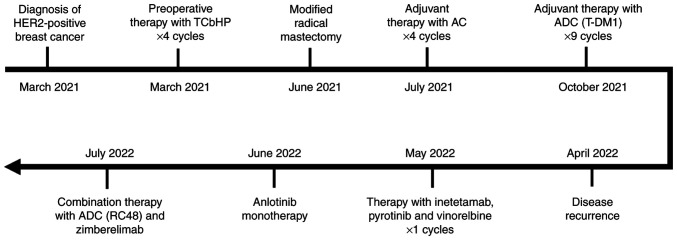
Whole treatment process before combination therapy with RC48 and zimberelimab. AC, doxorubicin liposome and cyclophosphamide; ADC, antibody drug conjugate; HER2, human epidermal growth factor receptor 2; RC48, disitamab vedotin; TCbHP, paclitaxel liposome, carboplatin, trastuzumab and pertuzumab; T-DM1, trastuzumab emtansine.

The patient received preoperative systemic treatment with 270 mg paclitaxel liposome on day 2, 500 mg carboplatin on day 3, trastuzumab on day 1 (first cycle at 8 mg/kg and subsequent cycles at 6 mg/kg) and pertuzumab on day 1 (first cycle at 840 mg and subsequent cycles at 420 mg), intravenously (IV), every 3 weeks (Q3W) (TCbHP) for four cycles (Fig. 1). However, in June 2021, MRI indicated that the left breast lesions and axillary nodes were progressively enlarged compared with the previous imaging scan. The patient received the last dose of trastuzumab and pertuzumab, and then underwent modified radical mastectomy later in June 2021. The postoperative pathology demonstrated that the tumor was 7.0×4.0×3.8 cm in size, with a negative margin, positive axillary nodes (2/17), and lymphovascular and nerve invasion. IHC indicated the following: ER 2+, 30%; PR-; HER2 1+; Ki-67, 40%; P53+; and EGFR+. Between July and October 2021, the patient received adjuvant therapy with 20 mg doxorubicin liposome on day 1, 40 mg on day 2 and 1,000 mg cyclophosphamide on day 1, IV, Q3W (termed AC) for four cycles. From October 2021, the patient was recommended to receive maintenance treatment with T-DM1 (200 mg; IV; Q3W) for nine cycles and underwent radiotherapy simultaneously at another institute (details unavailable) (Fig. 1).

In April 2022, the patient complained of new nodules in the left chest wall and following this, disease recurrence of breast cancer was pathologically diagnosed by needle biopsy (TNM classification: cT4N3M1) (Fig. S1C and D). The IHC results were as follows: ER- (Fig. S2C and D); PR- (Fig. S4C and D); HER2 2+; Ki-67, 60%; and PD-L1- (CPS=0) (anti-PD-L1 clone 22C3) (Fig. S5). Fluorescence *in situ* hybridization (FISH), performed according to the manufacturer's instructions (HER2 spectrum Orange/CEP 17 spectrum Green Probe; PathVysion HER-2 DNA probe kit; cat. no. 02J01-030; Abbott Molecular Diagnostics), indicated that there was no HER2 amplification, per the American Society of Clinical Oncology guideline (Fig. S6) (15). In May 2022, the patient received combination treatment with inetetamab (anti-HER2 antibody) plus pyrotinib and vinorelbine. After the first cycle of therapy, the patient discontinued the treatment because they wanted to join an ongoing clinical trial and then received anlotinib monotherapy, which was permitted during the screening period per subject inclusion criteria. Finally, the patient failed to be enrolled for the clinical trial and was admitted with chest wall recurrence of the left breast cancer in July 2022 (Fig. 1).

**Figure 2.**
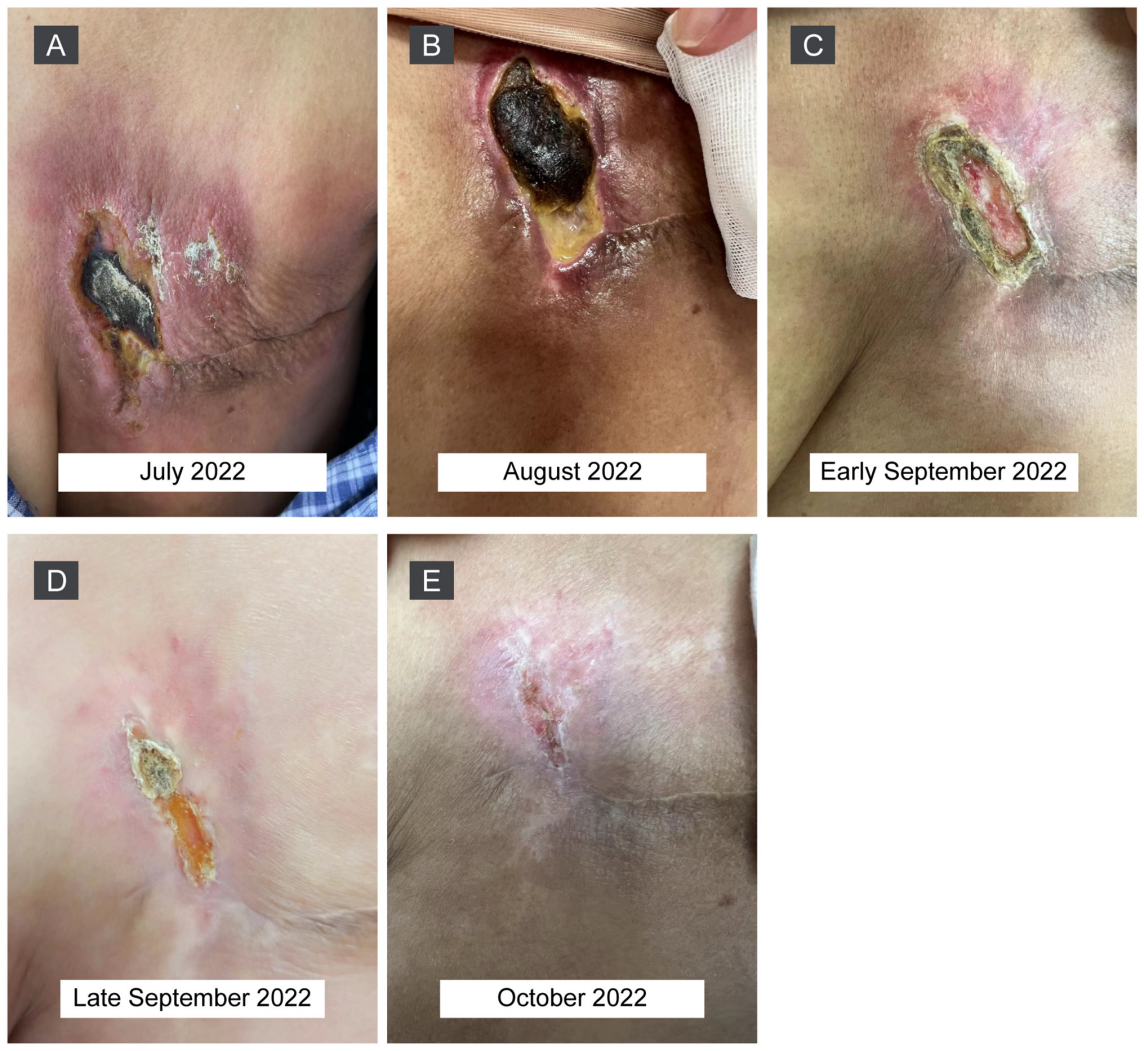
Changes in the tumor lesion on the surface of the left chest wall from the first dose of combination therapy with disitamab vedotin and zimberelimab (every 2 weeks). (A) Tumor manifestations on day 3 after the first dose of combination therapy. (B) Tumor involved areas were slightly reduced after two cycles of combination therapy. (C) Tumor lesion became markedly smaller after four cycles of combination therapy. (D) Tumor lesions continuously shrunk and healed before sixth dose. (E) Tumor lesions almost disappeared after six cycles of combination therapy.

Physical examination demonstrated that the tumor lesions were progressively enlarged and became confluent with the surface of the left chest wall (Fig. 2A). Combination therapy with RC48 (120 mg; day 1) and zimberelimab (240 mg; day 2), IV, every 2 weeks, was initiated. After two cycles of treatment, the skin affected by the tumor improved slightly (Fig. 2B) and thus, the treatment regimen was continued. Notably, the tumor lesions shrunk markedly after four cycles of treatment (Fig. 2C), healed gradually before the sixth dose (Fig. 2D) and almost disappeared after six cycles (Fig. 2E). The patient has remained in remission for >9 months as confirmed using chest enhanced-CT scans by the latest follow-up in March 2023 (Fig. S7) and has not experienced any immune-related adverse events with good tolerance. The next assessment of tumor response by imaging is planned to be performed in June 2023.

## Discussion

For recurrent metastatic HER2-positive breast cancer, the standard systemic therapies are well established in front-line settings and several choices of third-line treatment are also currently available (7). However, it remains a challenge to determine the regimens for patients who respond poorly to several prior perioperative systemic treatments, including chemotherapies and HER2-targeted therapy. In addition, during the course of HER2-positive breast cancer, a change in tumor expression of HER2 is associated with poor survival outcome and makes it difficult to select an optimal systemic therapy for either the adjuvant or recurrent metastatic setting (17–21). In previous years, HER2-targeted ADCs have become a novel option for recurrent/metastatic HER2-positive and low-expressive breast cancer (5,22,23). However, there are very limited data regarding the efficacy of combination therapy of ADCs and ICIs in heavily treated patients, particularly in those who do not respond to ADC agents.

In the present case, the patient had breast cancer with an initial tumor stage of T4N3M0, and their HER2 tumor expression of at diagnosis and at time of recurrence was IHC (3+) and IHC (2+)/FISH-negative, respectively. Therefore, preoperative therapy with TCbHP was recommended for the patient, which is the preferred regimen of preoperative therapy for this condition according to breast cancer guidelines (24,25). However, the tumor lesions were markedly enlarged after four cycles of treatment with TCbHP and the patient had to undergo surgery due to limited alternatives for preoperative treatment.

For HER2-positive breast cancer, as recommended by Chinese guidelines (24), AC followed by taxane, trastuzumab and pertuzumab (THP) is the preferred regimen of adjuvant therapy for patients with positive axillary lymph nodes. The patient completed post-surgical systemic therapy with four cycles of the AC regimen. Considering the poor tumor response to TCbHP treatment, T-DM1 instead of THP was used as part of the post-surgical treatment following the AC regimen. T-DM1 is also a recommended option for adjuvant therapy in patients with HER2-positive breast cancer according to the guidelines (24,25), based on the KATHERINE phase III study demonstrating that T-DM1 is a superior choice of adjuvant therapy for invasive HER2-positive breast cancer compared with trastuzumab (26).

The cancer unexpectedly recurred in the chest wall within 6 months of initiation with T-DM1 therapy. The protein expression was ER (−), PR (−), HER2 IHC (2+) (FISH-negative) and PD-L1 (−) (CPS=0) in recurrent lesions. Chinese guidelines (24) recommend pyrotinib (a HER2-targeted inhibitor) plus chemotherapy as a choice of salvage treatment regimens for HER2-positve breast cancer if prior treatment with trastuzumab has failed. In addition, inetetamab (an anti-HER2 antibody) plus vinorelbine has been approved in China for previously treated metastatic HER2-positive breast cancer. Hence, combination therapy with pyrotinib, inetetamab and vinorelbine was used as the first-line treatment for this patient.

However, the patient did not respond to the first line systemic therapy for recurrent disease. The tumor appeared to be resistant/refractory to multiple conventional chemotherapies, anti-HER2 antibodies and molecules, T-DM1 and radiation. Treatment with another ADC (RC48) in combination with zimberelimab was attempted, although the PD-L1 expression in the tumor was negative. RC48, instead of trastuzumab deruxtecan (T-DXd), was selected because T-DXd is currently unavailable in the region. RC48 plus zimberelimab was observed to exhibit marked antitumor activity for recurrent disease and has been achieving satisfactory PFS in the present case.

It was hypothesized that there were dual mechanisms contributing to the antitumor effect of the combination therapy. Compared with T-DM1, RC48 enhances antitumor activity through significant bystander effects, although they target the same molecule with the same class of cytotoxic agent (27). Preclinical data demonstrated that RC48 was more effective than T-DM1 in a trastuzumab- and lapatinib-resistant breast cancer model (28). Clinical data of RC48 also demonstrated its promising efficacy in HER2-low breast cancer, whereas T-DM1 did not exhibit its effect in these patients until now (29). Furthermore, ICIs and ADCs may have potential synergistic effects on tumor control by blocking the upregulated immune inhibitory pathway (e.g., PD-1/PD-L1 interaction) upon treatment with ADCs. In HER2-expressing breast tumor treated with ADCs, pre-clinical mouse model studies revealed that the expression of PD-1 in CD8^+^ T cells and of PD-L1 on tumor cells/tumor-associated macrophages is upregulated and tumor-infiltrating lymphocytes were also revealed to be increased compared with in the vehicle control. Therefore, ADCs plus anti-PD-1 antibodies were able to enhance the antitumor activities and prolong the survival time versus ADC monotherapy in mouse models (30,31). In addition, in a mouse model of HER2-positive tumors, combination treatment with RC48 and anti-PD-1 antibody was shown to be more effective than RC48 monotherapy in the tumor control (32). To the best of our knowledge, regarding ADCs plus PD-1/PD-L1 inhibitors in HER2-positive breast cancer, only one result from a phase II study (KATE2) has been reported and there is a lack of phase III data at present (33). The KATE2 study, which compared the efficacy of atezolizumab or placebo plus T-DM1 in HER2-positive advanced breast cancer, failed to show any meaningful clinical benefit on PFS; however, this may also be due to study limitations (e.g., small sample size, unblinded before target number of events reached) (34). Therefore, an ongoing phase III study (KATE3; NCT04740918) was designed to investigate the clinical benefit of this combination therapy (34). The combination of ADCs with PD-1 inhibitors appears to be a feasible approach for inoperable HER2-positive or low-expressive breast cancer to improve the efficacy and survival outcome; however, this is awaiting confirmation by further large-population clinical trials (33).

In the present case, there were three limitations in the diagnosis and anticancer treatment. Firstly, with the exception of PD-L1, other established biomarkers for immunotherapy (e.g., tumor mutation burden, microsatellite instability and mismatch repair deficiency) were not examined before initiation with zimberelimab. Secondly, adjuvant endocrine therapy for ER-positive/HER2-positive breast cancer was not implemented. Finally, there was a change in HER2 status between the primary and recurrent disease, but it has not been clarified whether the treatment regimen should be adjusted according to the discordance at present.

In conclusion, the present report describes the successful re-introduction of a HER2-targeted ADC combined with a PD-1 inhibitor in a patient with recurrent HER2-positive breast cancer whose disease progressed upon treatment with similar ADCs. The findings of this case suggest the potential clinical benefit of combination therapy with ICIs and ADCs in previously unsuccessfully treated patients with breast cancer showing HER2 upregulation, even after ADC failure. However, this should be further investigated in prospective clinical trials.

## Supplementary Material

Supporting Data

Supporting Data

## Data Availability

The datasets used and/or analyzed during the current study are available from the corresponding author on reasonable request.
