# Supplementary material for: Combination therapy with antibody‑drug conjugate RC48 (disitamab vedotin) and zimberelimab (PD‑1 inhibitor) successfully controlled recurrent HER2‑positive breast cancer resistant to trastuzumab emtansine: A case report
Source: Oncol Lett. 2023 Jul 5;26(2):359. doi: 10.3892/ol.2023.13945 (PMC10398622; doi:10.3892/ol.2023.13945)

Figure S1. H&E staining. H&E staining at (A) magnification x40 and (B) magnification x200 indicated invasive breast cancer using needle biopsy at diagnosis. H&E staining at (C) magnification x40 and (D) magnification x200 indicated invasive breast cancer using needle biopsy at recurrence. H&E, hematoxylin and eosin.

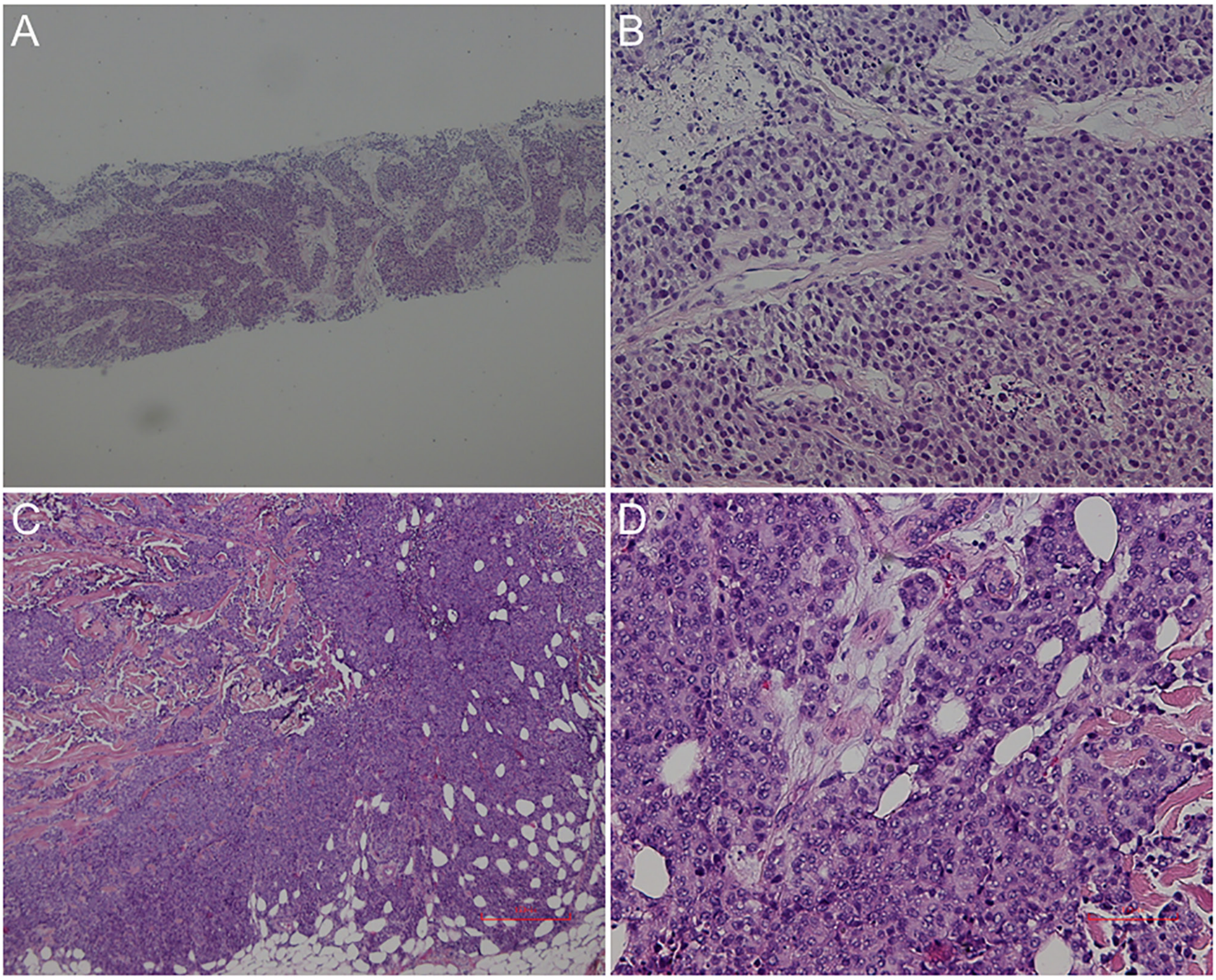

Figure S2. ER IHC staining. IHC staining at (A) magnification x40 and (B) magnification x200 showed breast cancer tissue with ER positivity (40%) at diagnosis. IHC staining at (C) magnification x40 and (D) magnification x200 showed breast cancer tissue with ER negativity at recurrence. ER, estrogen receptor; IHC, immunohistochemical.

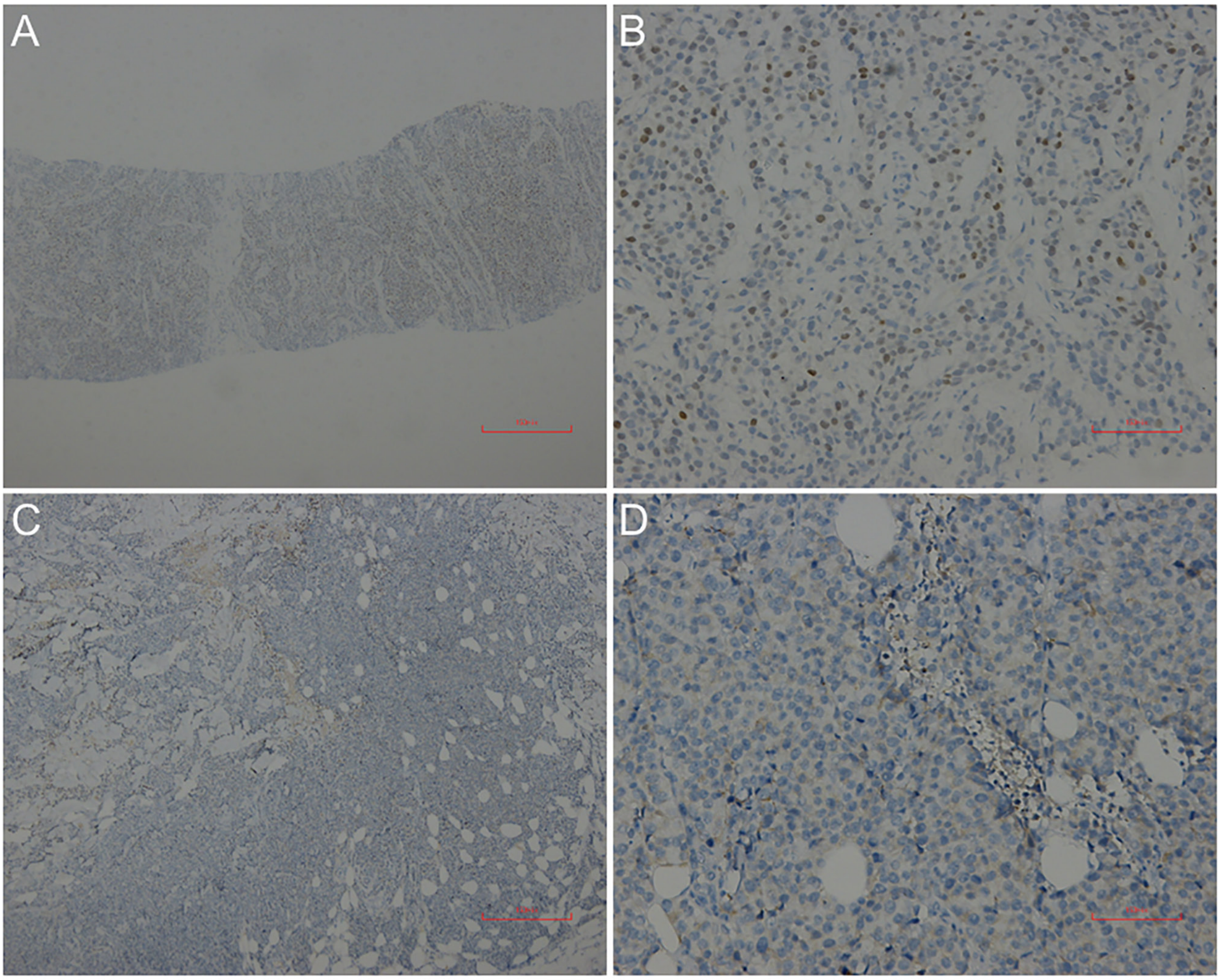

Figure S3. PR IHC staining. IHC staining at (A) magnification x40 and (B) magnification x200 showed breast cancer tissue with PR negativity at diagnosis. IHC staining at (C) magnification x40 and (D) magnification x200 showed breast cancer tissue with PR negativity at recurrence. IHC, immunohistochemical; PR, progesterone receptor.

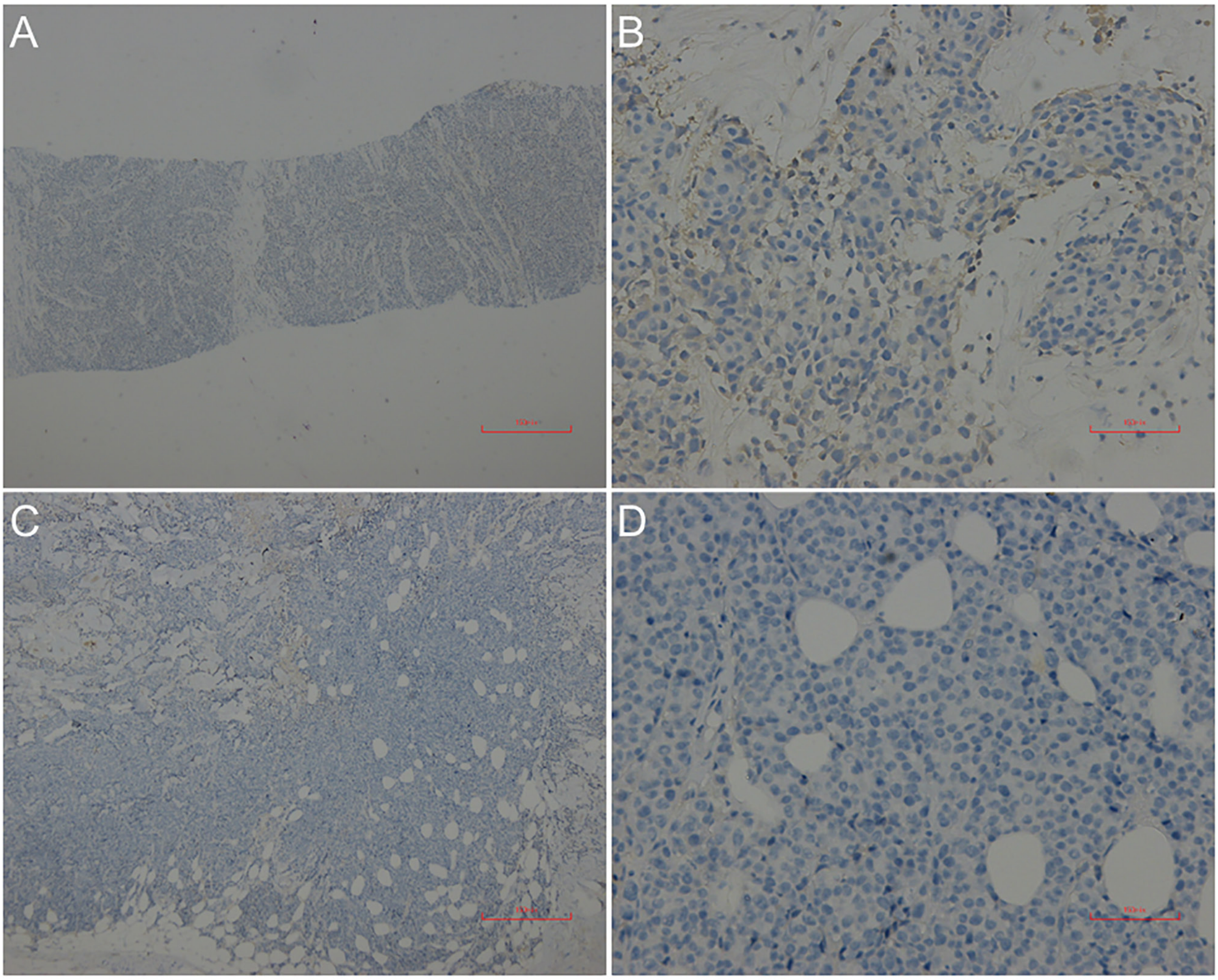

Figure S4. HER2 IHC staining. IHC staining at (A) magnification x40 and (B) magnification x200 showed breast cancer tissue with HER2 3+ at diagnosis. IHC staining at (C) magnification x40 and (D) magnification x200 showed breast cancer tissue with HER2 2+ at recurrence. HER2, human epidermal growth factor receptor 2; IHC, immunohistochemical.

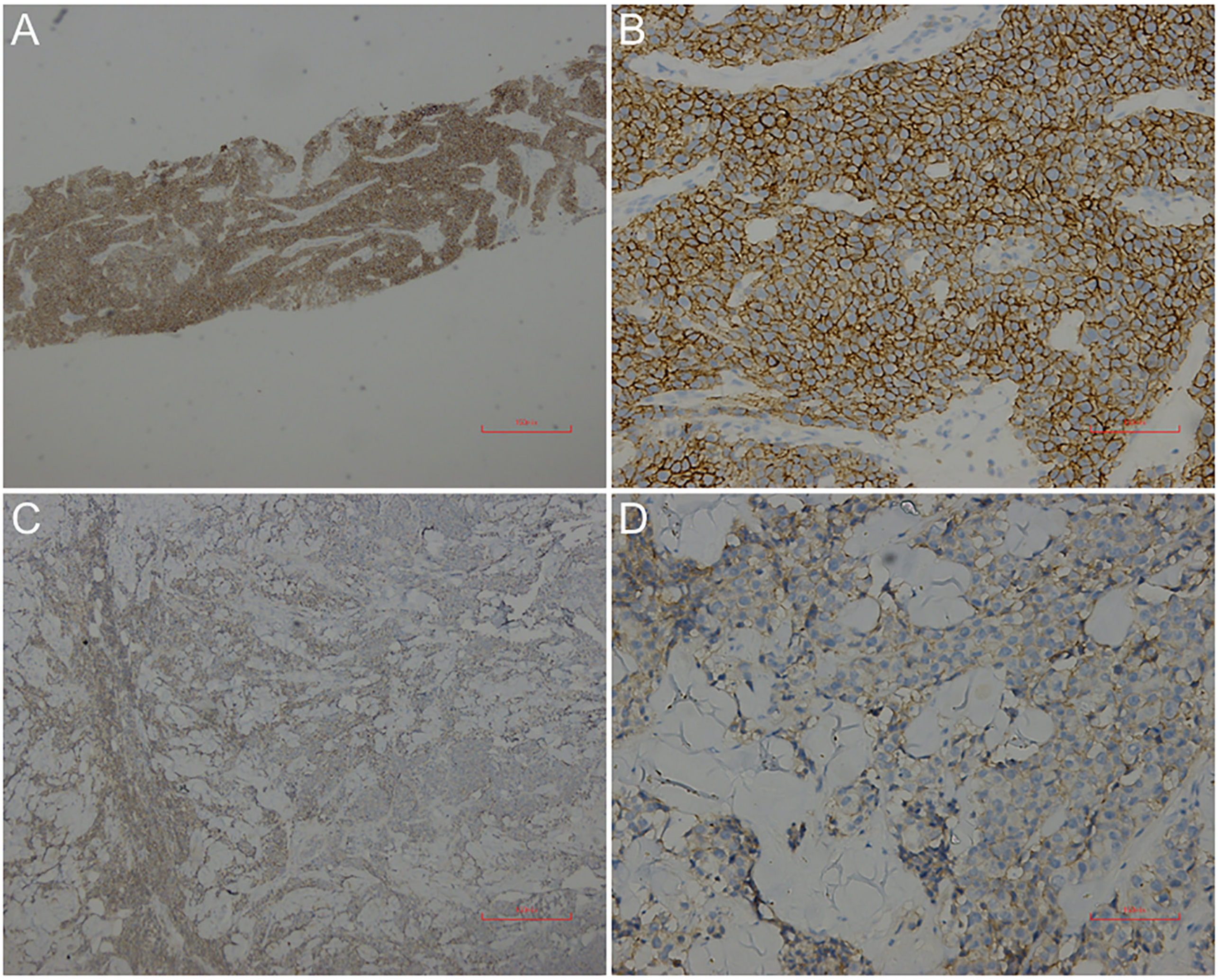

Figure S5. PD-L1 IHC staining at recurrence. IHC staining at (A) magnification x40 and (B) magnification x200 showed breast cancer tissue with PD-L1 negativity using needle biopsy at recurrence. IHC, immunohistochemical; PD-L1, programmed death ligand 1.

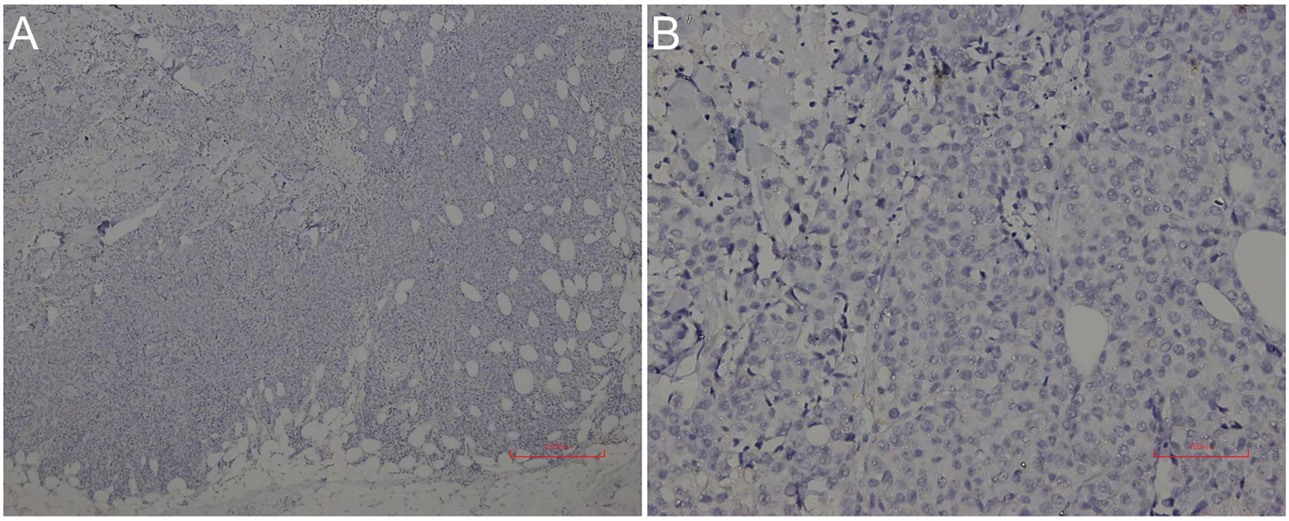

Figure S6. Fluorescence *in situ* hybridization indicated breast cancer tissue without amplification of human epidermal growth factor receptor 2 using needle biopsy at recurrence.

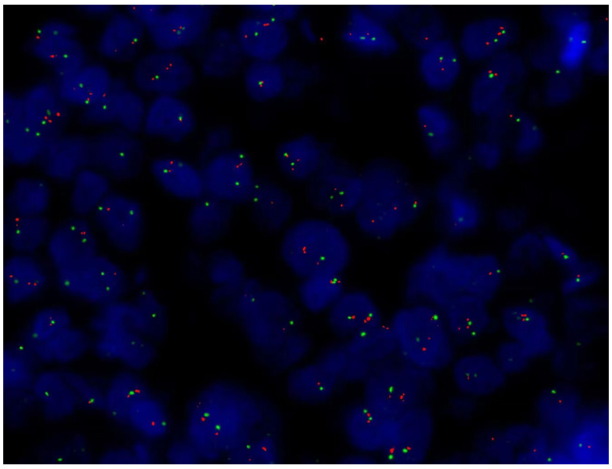

Figure S7. Findings of chest enhanced-CT scan. (A) CT scans indicated left breast cancer before surgery. (B) CT scans showed no recurrence observed in chest wall 3 months after surgery. (C) CT scans showed skin thickening in left chest with tumor recurrence 11 months after surgery. (D) CT scans showed tumor remission 3 months after combination treatment with RC48 and zimberelimab for recurrent disease. (E) CT scans showed tumor remission 7 months after combination treatment with RC48 and zimberelimab for recurrent disease. CT, computed tomography.

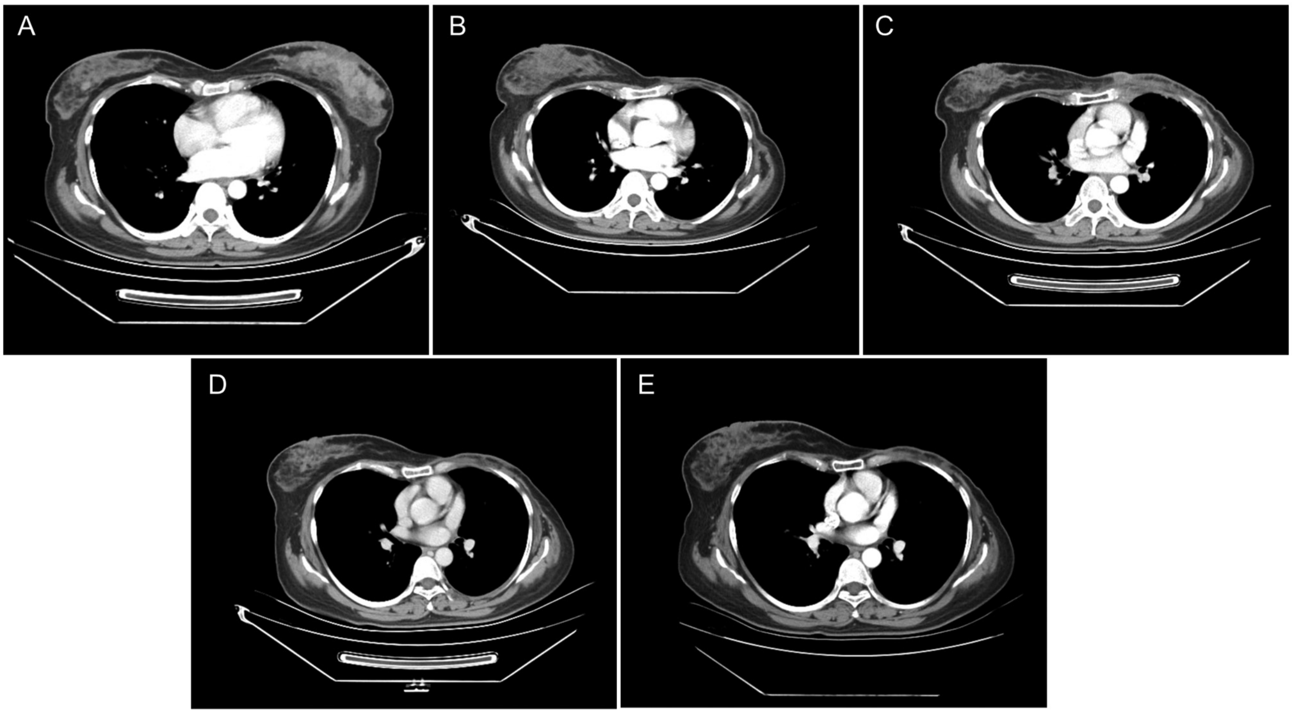

Supplement: Supporting Data [file Supplementary_Data1.pdf]
