# Supplementary material for: Combination therapy with antibody‑drug conjugate RC48 (disitamab vedotin) and zimberelimab (PD‑1 inhibitor) successfully controlled recurrent HER2‑positive breast cancer resistant to trastuzumab emtansine: A case report
Source: Oncol Lett. 2023 Jul 5;26(2):359. doi: 10.3892/ol.2023.13945 (PMC10398622; doi:10.3892/ol.2023.13945)
Supplement: Supporting Data [file Supplementary_Data2.pdf]

Table SI. Antibodies used for immunohistochemistry.

Antibody	Cat. no.	Supplier
Anti-ER (SP1)	790-4325	Roche Tissue Diagnostics
Anti-PR (1E2)	790-4296	Roche Tissue Diagnostics
Anti-HER2/neu (4B5)	790-4493	Roche Tissue Diagnostics
Anti-PD-L1 (22C3 pharmDx)	SK006	Agilent Technologies, Inc.

ER, estrogen receptor; PR, progesterone receptor; HER2, human epidermal growth factor receptor 2; PD-L1, programmed cell death protein-1 ligand.
